# Evaluation of an adapted version of the Diabetes Prevention Program for low- and middle-income countries: A cluster randomized trial to evaluate “Lifestyle Africa” in South Africa

**DOI:** 10.1371/journal.pmed.1003964

**Published:** 2022-04-15

**Authors:** Delwyn Catley, Thandi Puoane, Lungiswa Tsolekile, Ken Resnicow, Kandace K. Fleming, Emily A. Hurley, Joshua M. Smyth, Frank T. Materia, Estelle V. Lambert, Mara Z. Vitolins, Naomi S. Levitt, Kathy Goggin

**Affiliations:** 1 Center for Children’s Healthy Lifestyles and Nutrition, Children’s Mercy Kansas City, Kansas City, Missouri, United States of America; 2 University of Missouri-Kansas City School of Medicine, Kansas City, Missouri, United States of America; 3 University of the Western Cape School of Public Health, Cape Town, South Africa; 4 University of Michigan School of Public Health, Ann Arbor, Michigan, United States of America; 5 Life Span Institute, University of Kansas, Lawrence, Kansas, United States of America; 6 Health Services and Outcomes Research, Children’s Mercy Hospitals and Clinics, Kansas City, Missouri, United States of America; 7 College of Health and Human Development, Penn State University, University Park, Pennsylvania, United States of America; 8 UCT Research Centre for Health through Physical Activity, Lifestyle and Sport (HPALS), Division of Research Unit for Exercise Science and Sports Medicine, Faculty of Health Sciences, University of Cape Town, Cape Town, South Africa; 9 Department of Epidemiology & Prevention, Wake Forest School of Medicine, Winston-Salem, North Carolina, United States of America; 10 Department of Medicine and Chronic Disease Initiative for Africa, Faculty of Health Sciences, University of Cape Town, Cape Town, South Africa; 11 University of Missouri-Kansas City School of Pharmacy, Kansas City, Missouri, United States of America; Shanghai Jiao Tong University Affiliated Sixth People’s Hospital, CHINA

## Abstract

**Background:**

Low- and middle-income countries (LMICs) are experiencing major increases in diabetes and cardiovascular conditions linked to overweight and obesity. Lifestyle interventions such as the United States National Diabetes Prevention Program (DPP) developed in high-income countries require adaptation and cultural tailoring for LMICs. The objective of this study was to evaluate the efficacy of “Lifestyle Africa,” an adapted version of the DPP tailored for an underresourced community in South Africa compared to usual care.

**Methods and findings:**

Participants were residents of a predominantly Xhosa-speaking urban township of Cape Town, South Africa characterized by high rates of poverty. Participants with body mass index (BMI) ≥ 25 kg/m^2^ who were members of existing social support groups or “clubs” receiving health services from local nongovernmental organizations (NGOs) were enrolled in a cluster randomized controlled trial that compared Lifestyle Africa (the intervention condition) to usual care (the control condition). The Lifestyle Africa intervention consisted of 17 video-based group sessions delivered by trained community health workers (CHWs). Clusters were randomized using a numbered list of the CHWs and their assigned clubs based on a computer-based random allocation scheme. CHWs, participants, and research team members could not be blinded to condition. Percentage weight loss (primary outcome), hemoglobin A1c (HbA1c), blood pressure, triglycerides, and low-density lipoprotein (LDL) cholesterol were assessed 7 to 9 months after enrollment. An individual-level intention-to-treat analysis was conducted adjusting for clustering within clubs and baseline values. Trial registration is at ClinicalTrials.gov (NCT03342274). Between February 2018 and May 2019, 782 individuals were screened, and 494 were enrolled. Participants were predominantly retired (57% were receiving a pension) and female (89%) with a mean age of 68 years. Participants from 28 clusters were allocated to Lifestyle Africa (15, *n* = 240) or usual care (13, *n* = 254). Fidelity assessments indicated that the intervention was generally delivered as intended. The modal number of sessions held across all clubs was 17, and the mean attendance of participants across all sessions was 61%. Outcome assessment was completed by 215 (90%) intervention and 223 (88%) control participants. Intent-to-treat analyses utilizing multilevel modeling included all randomized participants. Mean weight change (primary outcome) was −0.61% (95% confidence interval (CI) = −1.22, −0.01) in Lifestyle Africa and −0.44% (95% CI = −1.06, 0.18) in control with no significant difference (group difference = −0.17%; 95% CI = −1.04, 0.71; *p* = 0.71). However, HbA1c was significantly lower at follow-up in Lifestyle Africa compared to the usual care group (mean difference = −0.24, 95% CI = −0.39, −0.09, *p* = 0.001). None of the other secondary outcomes differed at follow-up: systolic blood pressure (group difference = −1.36; 95% CI = −6.92, 4.21; *p* = 0.63), diastolic blood pressure (group difference = −0.39; 95% CI = −3.25, 2.30; *p* = 0.78), LDL (group difference = −0.07; 95% CI = −0.19, 0.05; *p* = 0.26), triglycerides (group difference = −0.02; 95% CI = −0.20, 0.16; *p* = 0.80). There were no unanticipated problems and serious adverse events were rare, unrelated to the intervention, and similar across groups (11 in Lifestyle Africa versus 13 in usual care). Limitations of the study include the lack of a rigorous dietary intake measure and the high representation of older women.

**Conclusions:**

In this study, we found that Lifestyle Africa was feasible for CHWs to deliver and, although it had no effect on the primary outcome of weight loss or secondary outcomes of blood pressure or triglycerides, it had an apparent small significant effect on HbA1c. The study demonstrates the potential feasibility of CHWs to deliver a program without expert involvement by utilizing video-based sessions. The intervention may hold promise for addressing cardiovascular disease (CVD) and diabetes at scale in LMICs.

**Trial registration:**

ClinicalTrials.gov NCT03342274.

## Abstract 2

In a cluster randomized trial, Delwyn Catley and colleagues evaluate an adapted version of the Diabetes Prevention Program in South Africa.

## Author summary

**Why was this study done?:**

- Low- and middle-income countries (LMICs) are experiencing high rates of chronic diseases such as diabetes and cardiovascular conditions linked to overweight and obesity.
- “Lifestyle” programs developed in high-income countries that help participants to lose weight are effective at preventing and reducing chronic disease but require adaptation to be feasible and effective in LMICs.

**What did the researchers do and find?:**

- This study evaluated whether a lifestyle program (Lifestyle Africa*)* adapted for overweight participants in an underresourced community in South Africa could improve upon usual care in reducing participants’ weight and improving other indicators of health.
- Overweight volunteers from 28 preexisting “health clubs” receiving health services from community health workers (CHWs) were randomly allocated to Lifestyle Africa (15 clubs, *n* = 240) or usual care (13 clubs, *n* = 254).
- Participants in the Lifestyle Africa program participated in regular group sessions to learn strategies for weight loss through healthy eating and physical activity.
- Participants’ weight, blood pressure, cholesterol, and hemoglobin A1c (HbA1c; an indicator of mean blood sugar over 3 months and related to diabetes status) were assessed at the beginning of the trial and 7 to 9 months later.
- Results revealed no effect of the intervention on the main outcome of weight loss (mean weight reduction was just 0.17% greater in Lifestyle Africa), blood pressure, or cholesterol. However, the intervention resulted in lower HbA1c (the mean was 0.24 lower in Lifestyle Africa).

**What do these findings mean?:**

- The results indicate that Lifestyle Africa was feasible for CHWs to deliver, and the significant effect on HbA1c suggests that the intervention may have potential for addressing chronic disease at scale in LMICs.
- However, the relatively small effect on HbA1c and lack of effect on other outcomes indicate that the intervention needs to be strengthened before being recommended for widescale use.
- The study was limited by lack of an accurate measure of any dietary changes among participants as well as the high number of older females who may have respond in a unique way to the intervention.

## Introduction

Approximately 15 million premature deaths per year worldwide are attributable to noncommunicable diseases, including cardiovascular diseases (CVDs) and diabetes mellitus [1]. Over 85% of these deaths occur in low- and middle-income countries (LMICs), where the disproportionate burden of noncommunicable disease is expected to increase [2]. In South Africa, noncommunicable diseases are more prevalent than HIV/AIDS and tuberculosis combined, with CVD being the leading category [3]. The prevalence of type 2 diabetes mellitus (T2DM) in South Africa is estimated to be about 15% with higher rates among women and older people [4].

The increase in CVD and T2DM in LMICs is attributed partly to rising incomes and urbanization, which leads to a shift from eating unrefined carbohydrates to greater intake of fats, sweeteners, and animal source foods, as well as highly processed foods [5]. Urbanization is also linked to reductions in physical activity [6]. Cultural beliefs and practices (e.g., less stigmatization of obesity and a preference for being overweight) may also play a role [7].

In high-income countries, lifestyle interventions such as the US Diabetes Prevention Program (DPP) have been developed to combat unhealthy changes in diet and physical activity [8]. In the program “lifestyle coaches,” who are usually health professionals such dietitians, nutritionists, or counselors, encourage participants to engage in at least 150 minutes of moderate physical activity per week and to reduce body weight by 7% over 6 months. Randomized trials have demonstrated powerful clinical effects in preventing the development of T2DM [8,9] as well as reducing weight and improving glucose control, blood pressure, triglycerides, and high-density lipoprotein (HDL) cholesterol among individuals with T2DM [10].

Similar lifestyle programs targeting weight change through both physical activity and diet have also been evaluated in randomized trials in some lower income countries including in Thailand [11], Brazil [12], and China [13], with generally positive health effects [14]; however, these interventions were delivered by researchers or high-level health professionals and mostly through health clinics or work sites. The reach of lifestyle interventions in LMICs will be greatly limited if they depend on scarce and expensive professional healthcare workers and if participant time and travel costs are barriers to access. Studies in the US have shown that lay or community health workers (CHWs) can be successfully deployed as DPP interventionists [15], but randomized trials in LMICs are lacking. Two notable exceptions are the Kerala DPP in India [16] and the Grenada Heart Project [17], both of which used a peer support model of intervention. Both studies found some positive effects on composite health risk indicators, but neither study found significant effects on key clinical indicators such as weight, hemoglobin A1c (HbA1c), or blood pressure.

To extend this work, we developed a new version of the DPP (“Lifestyle Africa”) tailored specifically for an underresourced community in South Africa and designed to be delivered by CHWs. South Africa has a long history of employing CHWs as key interventionists in its national primary care program where they connect community members with services provided in local health facilities [18]. The purpose of our study was to test the adapted program in a cluster randomized trial with CHW teams and their associated clubs as clusters. The primary outcome analysis compared percentage weight loss between Lifestyle Africa and usual care participants. HbA1c, blood pressure, triglycerides, and low-density lipoprotein (LDL) cholesterol were selected as secondary outcomes because prior studies of lifestyle interventions have more typically reported changes in weight [9,19].

## Methods and analysis

### Methods

#### Study design and participants

This study was a 2-arm, parallel group, cluster-designed randomized controlled trial with a planned follow-up and crossover of the control arm after 1 year. Participants were enrolled in 2 waves separated by a year. Although the goal was to conduct a 1-year follow-up, we chose to avoid conducting our assessment after the long annual December to January holiday break in which most community members and CHWs travel to rural areas. We believed the disruption of routine and session attendance had the potential to confound our assessment. Enrollment and baseline assessment therefore took place as early as possible in the calendar year (February and March), and the main outcome assessment time point was as late as possible in the same calendar year (i.e., mid-September through early December) resulting in follow-up taking place approximately 7 to 9 months after enrollment. Mean, median, and interquartile range of follow-up was 210, 212, and 28.5 days for the control group and 224, 224, and 21.5 days for the Lifestyle Africa group. This report focuses on our primary and secondary biologic outcomes at the first follow-up. Full details of the study are described elsewhere [20,21]. The study was approved by the ethics committees at the University of Cape Town (primary; number 109/2017) and Children’s Mercy Kansas City (number 15080328) and is registered with ClinicalTrials.gov, number NCT03342274. This study is reported per the Consolidated Standards of Reporting Trials (CONSORT) guidelines (S1 Checklist).

The study setting was a predominantly Xhosa-speaking urban township of Cape Town, South Africa characterized by high rates of poverty and unemployment, low education, and high rates of overweight and obesity [22,23]. Our partner NGOs were utilizing CHWs to support delivery of basic healthcare services (e.g., medication delivery and wellness programs), and CHWs had received basic training in in-home–based care, chronic disease management, and wellness. In consultation with their CHWs, our partner NGOs adopted the Lifestyle Africa Program as their standard of care (training and oversight described below). CHWs varied in their educational attainment but had sufficient reading, writing, and arithmetic skills to maintain attendance registers and medication logs. CHW services were being provided through community clubs that met at community facilities (e.g., churches) or homes.

To reach our recruitment goal, we planned and conducted recruitment at all clubs in one branch of one partner NGO and all clubs from the other partner NGO. Two scripted video-based recruitment sessions were held at club meetings to introduce the study to members. Recruitment sessions were led by either CHWs or research team members. Members were invited to attend a club enrollment session held at a convenient location. At the enrollment session, participants completed written informed consent, eligibility screening, and baseline assessment. To serve as many club members as possible, that criterion for inclusion was limited to being overweight or obese (i.e., BMI ≥ 25 kg/m^2^). There was no exclusion related to diabetes diagnosis. If a club member’s BMI could not be established because a physical disability prevented the assessment of weight the individual was not included. Exclusion criteria were the following: (1) having a blood pressure >160 (systolic) and/or >100 mm Hg (diastolic) [24]; (2) very high blood sugar (HbA1c > 11%) [19]; (3) pregnant, breastfeeding, or planning to become pregnant; (4) use of oral steroid medications that may affect weight loss; (5) not planning to stay in the club over the next 2 years; and/or (6) exhibiting intellectual disabilities that would prevent necessary understanding of the program requirements.

#### Randomization and masking

Clusters constituted “CHW teams” and their associated clubs because CHWs worked individually, in pairs, and occasionally in trios with a particular club. CHW teams were stratified within NGOs and allocated using a 1:1 scheme to Lifestyle Africa or usual care. Clusters were randomized by the statistician (KF) using a numbered list of the CHWs and their assigned clubs based on a computer-based random number generated allocation scheme. Clusters were randomized prior to participant enrollment (and hence prior to individual consent) in order to establish which CHWs needed to be trained to deliver the intervention. The nature of the trial precluded blinding of CHWs, study staff, or participants.

#### Lifestyle Africa intervention

The Lifestyle Africa intervention was adapted from the US Centers for Disease Control National DPP [25]. The US DPP is a group-based behavioral intervention program to encourage physical activity and dietary changes that will lead to clinically meaningful weight loss. Session content is broadly based on social cognitive and problem-solving theory principles of behavior change and encompass topics such as goal setting, self-monitoring of physical activity of diet, healthy nutrition, stress management, and stimulus control [26]. During group sessions, participants receive informational content as well as mutual support through the opportunity to discuss their individual challenges and successes. The program is typically delivered by health professionals with training in nutrition or counseling. Preliminary work involved formative research conducted in collaboration with 2 community advisory boards [27]. The community advisory boards informed the development of the program and the design of the training manual and curriculum. Program development also included demonstration of session feasibility in pilot testing [28].

The Lifestyle Africa intervention retained the DPP goals for participants of 7% of weight loss and 150 min/week of physical activity and key elements from the 16 “core” and 15 “postcore” DPP sessions [25,26], while integrating necessary cultural and language adaptations relevant for the local community. For example, intervention content was adapted to explain the connection between chronic disease and lifestyle in accessible language. Culturally and literacy-adapted methods for calculating the number of physical activity minutes and the number of calories consumed were devised. The number of core sessions was also increased to 17 to reduce the amount of information covered in some of the sessions.

The primary Lifestyle Africa adaptation was to allow the program to be facilitated by CHWs rather than highly trained clinician facilitators with expert knowledge related to diet, physical activity, and behavior change. To make this feasible, CHWs were trained to provide participants with core intervention content via a video-based curriculum that closely followed the session content of the original DPP. To cover the content without excessive session length, an additional session was created, resulting in 17 rather than 16 sessions. Lifestyle Africa session videos were developed in the Xhosa language and featured a first-language Xhosa-speaking narrator in conjunction with animation and photos. Pauses were built into the videos when CHWs engaged participants with questions and activities (e.g., completing worksheets that personalized and reinforced concepts). Participants received a program book in either Xhosa or English (if preferred) with worksheets, information, and forms needed for each program session (e.g., goal setting sheets). CHWs used a facilitator manual in which each Lifestyle Africa session video had an accompanying “session guide” with step-by-step instructions and scripts for facilitating each of the 17 core program sessions. In addition, 12 similar but briefer “postcore” sessions were developed without video content. CHWs delivered the program weekly (or sometimes biweekly to accommodate a club’s meeting schedule), although necessary adjustments were made to allow for circumstances when clubs could not meet (e.g., holidays, pension collection days, and neighborhood protests). Clubs that completed the 17 core sessions continued with monthly postcore meetings until the follow-up assessment.

To enhance the efficacy of the program, text messaging (i.e., SMS) was employed to promote healthy lifestyle modifications and behavior change [29]. A text messaging system sent 2 texts per day (late morning and evening) to participants’ mobile phones and provided program reminders, fostered self-efficacy and motivation, affirmed behavior change efforts, and aided in implementation planning (e.g., healthy lifestyle tips such as “changing the way you stay active will help keep exercising fun”). The same texts were delivered to all participants, with weekly message content adapted over time to refer to the content of the session the participant had most recently attended.

CHW training included 3 days of didactic interactive workshops addressing group facilitation, diabetes management, behavior change theory, and motivational interviewing (MI). This was followed by 8 weekly half-day sessions of experiential training as mock Lifestyle Africa participants in which they participated in every core session and took turns practicing all key facilitation activities and skills (e.g., conducting the weighing in of participants, setting up the projector and using the video, and facilitating the opening discussion of the session). These trainings were provided by South African research team members in the Xhosa language (including LT and TP), who were themselves trained by authors DC, KG, and KR.

CHWs were supervised by NGO managers in accord with their usual practice. Assistance was provided as needed by research team members who observed a subset of Lifestyle Africa sessions to ensure intervention fidelity. Research team members observed the first 10 sessions for each club and then about 1 session every 5 to 8 weeks. Team members used checklists to verify the extent of completion (not completed, partly completed, and completed) of specific session facilitation elements (e.g., set up and played the session videos successfully, completed the weigh-in, and followed the steps in the session script). They also completed an adapted version of the OnePass measure for MI competence [30] to rate the quality of group facilitation, MI skills, and overall performance on a 7-point Likert scale (1 = poor/never, 4 = good/often; and 7 = excellent/always). Ratings of 4 or higher were considered acceptable.

#### Usual care

In usual care, CHWs continued to lead clubs in their usual activities of approximately monthly monitoring of weight, blood pressure, and blood glucose and delivery of medication. These clinical assessments were not used in the study.

#### Enrollment and assessment

Enrollment and baseline assessment began with wave 1 in 2018. Follow-up assessments were held at a convenient location (e.g., the club facility or a nearby community hall) for club members and were conducted in the same fashion as the baseline assessment. If necessary, transportation to the assessment site was provided. A flyer with instructions to wear light clothing, to use the restroom, and to bring medications and their mobile phone number was provided before each assessment session. After completing written informed consent participants proceeded through each assessment station including the biometric assessments and interviewer-administered survey. Participants received a R150 (approximately $12 USD) gift voucher for completing each assessment.

### Outcomes

The primary outcome was percentage of weight lost between the baseline and the follow-up visit, measured at the individual level. Weight was measured to the half kilogram with a portable electronic platform scale (Seca 876) after removal of shoes and outer clothing. Height was measured at baseline for purposes of calculating body mass index (BMI), which was calculated as weight in kilograms divided by the square of height in meters. Secondary outcomes were HbA1c, nonfasting LDL cholesterol, triglycerides, and blood pressure. HbA1c and lipids were all measured via an automated capillary sample assay using an Afinion AS100 analyzer (Alere, Waltham, Massachusetts, USA), which has shown very good agreement with laboratory methods [31,32]. Blood pressure was determined with an Omron HBP1300 instrument (Omron Healthcare, Kyoto, Kyoto, Japan) [33]. Two measurements were obtained and, if either systolic or diastolic blood pressure measurement were discrepant by more than 5 mm Hg, a third measurement was taken. For analysis, the mean of the last 2 measurements taken was used. Participants brought all of their medications to the enrollment session to determine medication usage, the names of which were recorded by an interviewer. Demographic information including age, sex, approximate monthly income (from “no income” to “R204,801 or more” using increments that doubled at each step starting at R400-R800, R800-R1600, R1600-R3200, etc.), education (highest grade level reached from no formal schooling through any tertiary education), and relationship status (single; married; not married living with partner; separated; divorced; and widowed) was collected via interview and responses entered into a REDCap survey [34].

To monitor the trial and unexpected or severe adverse events, a Data Safety Monitoring Board oversaw the study and approved stopping rules related to serious adverse events and failure to recruit and retain participants. The board comprised members with expertise in public health, medicine, psychology, and statistics and operated independently from the study team and the funder. The board reviewed serious adverse events as well as enrollment, session completion, and follow-up rates in twice yearly meetings. Only serious adverse events and unanticipated problems were monitored due to the low-risk nature of the trial. In closed session, the board made decisions regarding continuation of the trial.

### Statistical analysis

Power analysis was based on an anticipated sample of 28 total clusters averaging 18 to 19 participants for a total N of 518. We assumed a treatment weight loss of 7.21% ± 0.57% (−7.1 kg) for intervention participants and 1% (<1.0 kg) weight loss for controls [35], yielding a conservative estimated effect size for weight loss of 0.37. Lindström and colleagues [36] reported reduction in HbA1c with an effect size of 0.31. Power analyses indicated that a trial with 28 randomly assigned clubs and 20 participants per club would provide >80% power to detect effect sizes of 0.27 with an intracluster correlation of 0.01 and 0.34 with an intracluster correlation of 0.05. Allowing for 25% attrition at the participant level (i.e., assuming 15 participants per cluster) and assuming intracluster correlation coefficients (ICC) between 0.01 and 0.05, we were adequately powered to detect effects between 0.31 and 0.37.

Our prespecified analysis plan is outlined in our published study protocol [20]. Subsequent to publication of the protocol the study sponsor (the National Institutes of Health) required the addition of analysis of outcomes by sex to our analysis plan. In accord with our prespecified plan, preliminary analyses examined baseline equivalence across arms to identify covariates for the main analyses. Prespecified multilevel modeling analyses were conducted using the MIXED procedure within SAS 9.4 (SAS Institute, Cary, North Carolina, USA). ICCs were calculated from empty models with no predictors. Initially, we examined intent-to-treat models that included occasions (baseline/year 1) nested within participants (Level 2), which were nested within clusters (Level 3). Planned examination of simple effects of condition at baseline revealed that there were not statistically significant differences between groups for any of our outcomes of interest. However, some outcomes had relatively small *p*-values (e.g., weight, *p* = 0.10), suggesting that analyses examining group differences at follow-up controlling for baseline levels would be appropriate. Thus, post hoc analyses examined models where participants (Level 1 units) were nested within clusters (Level 2 units) with outcomes at the end of year 1 as the dependent variable and baseline values as covariates.

For the primary outcome, percentage weight loss from baseline to the end of year 1 was the dependent variable in the multilevel models described above. Random intercepts were modeled across clusters. Similar models were evaluated for each of the secondary outcomes (blood pressure, HbA1c, LDL, and triglycerides) with outcomes at follow-up as the dependent variable and baseline level of the outcome as a covariate in each model. The first set of models included no covariates and only examined the effect of intervention. To explore any potential influence of participants baseline weight on outcomes, a second set of models was conducted, adjusting for baseline BMI. To explore the potential impact of medications we also reanalyzed any significant effects after removing participants on medications who improved in HbA1c. A third set of models were used for preplanned examination of the effect of intervention within sex groups by adding sex and sex by condition interactions to the model. Standardized effect sizes (Hedges’ *g*) were calculated using differences in the model adjusted means between the treatment arms divided by the pooled observed standard deviation at baseline. To better elucidate clinical effects, we conducted post hoc analyses for significant effects (*p* < 0.05) using established clinical cut points. We categorized participants according to whether they lowered (improved), maintained, or raised in clinical category from baseline and examined treatment arm differences utilizing multilevel logistic regression for clustered outcomes using SAS Proc GLIMMIX with a multinomial distribution and a cumulative logit link function.

In response to peer review, we conducted additional post hoc analyses. We examined potential bias related to loss to follow-up (i.e., participants for whom follow-up data could not be collected because participants died, left the area, or could not be located) by comparing baseline characteristics between those retained and those who were lost to follow-up within each arm. We also examined absolute weight while adjusting for absolute weight at baseline as this approach has been shown to provide more precise estimates than percentage weight loss [37]. Because of the inclusion of participants in different diabetes categories, we also conducted analyses of the effect of intervention on weight within diabetes category by adding diabetes category and diabetes category by condition interactions to the model. For significant effects, we conducted tipping point analyses to judge the effect of missing data.

## Results

**Fig 1:**
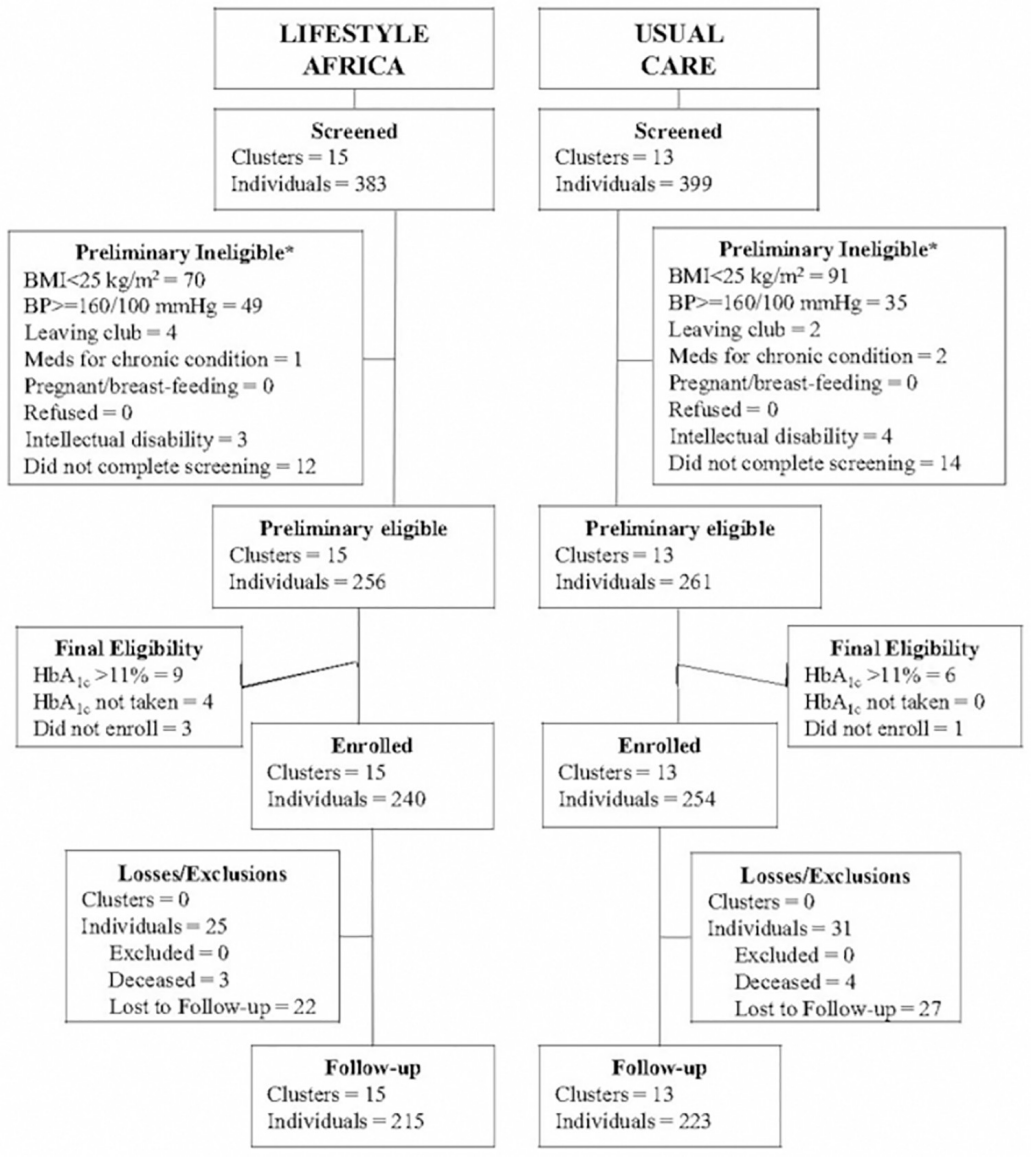
CONSORT flow diagram. *Participants could be ineligible for more than one reason. BMI, body mass index; BP, blood pressure; HbA1c, hemoglobin A1c; Meds, medications.

Between February 2018 and March 2019, we screened 782 potential participants in 28 clusters. Of these, 494 individuals were eligible and were enrolled (Fig 1). Main reasons for ineligibility were BMI too low (56% of those ineligible) and blood pressure too high (29% of ineligibles). At the follow-up, completed between September 2018 and December 2019, 438 participants (28 clusters) were assessed including 215 (90%) in Lifestyle Africa and 223 (88%) in usual care.

Table 1 shows the baseline characteristics of participants by arm, while Table 2 shows baseline and follow-up levels of biometric measurements and medication use by arm. Participants were majority women and had a mean age of 68 years. Baseline characteristics were similar across groups, with 8% of participants in both groups employed and 31% of participants in both groups in the lowest income category and 57% in the middle category, although educational attainment was somewhat higher in the usual care group (52% advancing beyond eighth grade versus 44% in Lifestyle Africa). Mean BMI was in the obese range for both groups, although the Lifestyle Africa group had a lower baseline BMI (33.8 versus 35.2 in usual care). Although baseline HbA1c was similar between groups, more participants in the usual care group were in the prediabetic range (53.1% versus 44.2%), while fewer were in the diabetic range (22.4% versus 28.8%). Comparison of baseline characteristics between those who completed the trial and those lost to follow-up within each arm revealed notable differences in weight, BMI, HbA1c, and sex within the Lifestyle Africa arm. At baseline, mean weight was 91.89 kg for those lost to follow-up and 82.95 kg for those who completed the study [*F*(1,464) = 6.10, *p* = 0.01]. Baseline BMI was 36.04 for those lost to follow-up and 33.54 for those who completed the study [*F*(1,464) = 3.10, *p* = 0.08]. Baseline HbA1c was 5.90 for those who were lost to follow-up and 6.41 for those who completed the study [*F*(1,464) = 4.40, *p* = 0.04]. With respect to sex, males represented a higher proportion among those lost to follow-up than those who completed the study [24% versus 10%, respectively; *F*(1,464) = 4.25, *p* = 0.04]. The only notable difference within the usual care arm was with respect to sex, with a higher proportion of those lost to follow-up being males [19% versus 10%; *F*(1,464) = 2.45, *p* = 0.12].

**Table 1:** Participant baseline characteristics.

	Lifestyle Africa (*n* = 240)	Usual care (*n* = 254)
Number of clusters	15	13
Cluster size	16.00 (6.50)	19.54 (4.50)
Cluster size range	4 to 29	11 to 30
Sex Male Female	28 (11.7%) 212 (88.3%)	28 (11.0%) 226 (89.0%)
Age (years)	67.6 (10.69)	67.8 (10.76)
Education attainment Below eighth grade Above eighth grade	135 (56.5%) 104 (43.5%)	118 (47.8%) 129 (52.2%)
Relationship status Single Married/living together Widowed/divorced/separated	60 (25.0%) 57 (23.8%) 123 (51.2%)	72 (29.1%) 59 (23.9%) 116 (47.0%)
Employment status Unemployed/home/scholar Employed Pensioner	88 (36.7%) 19 (7.9%) 133 (55.4%)	76 (30.9%) 20 (8.1%) 150 (61.0%)
Income (monthly) Less than R1,600 (<$126.10) R1,600 to R3,200 ($126.10 to $252.20) More than R3,200 (>$252.20)	74 (30.8%) 138 (57.5%) 28 (11.7%)	75 (30.5%) 140 (56.9%) 31 (12.6%)
Diabetes medication* Metformin Glimepiride Insulin	48 (20.2%) 24 (10.1%) 6 (2.5%)	50 (20.1%) 25 (10.0%) 7 (2.8%)
BMI	33.82 (6.24)	35.22 (7.13)

Data are *n* (%) or mean (SD). Rand to US Dollar exchange rate based on the mean of Feb 2018 and Feb 2019 rates. Counts do not add up to the group ns in all cases due to missing data; percentages are based on observed ns for that item.

*Diabetes medication categories are not mutually exclusive, i.e., some participants reported taking more than one.

BMI, body mass index.

**Table 2:** Raw data for biometrics and medication use at baseline and follow-up.

	Lifestyle Africa		Usual care	
	Baseline (*n* = 240)	Follow-up (*n* = 215)	Baseline (*n* = 254)	Follow-up (*n* = 223)
Weight (kg)	83.94 (16.54) 240	82.36 (15.90) 215	86.55 (17.79) 254	86.05 (18.28) 223
% Change in body weight	-	–0.66 (4.10) 215	-	–0.44 (3.61) 223
Blood pressure (mm Hg)^†^ Systolic Diastolic	126.15 (15.82) 240 74.18 (9.51) 240	129.51 (19.89) 214 74.29 (11.03) 214	126.18 (15.59) 254 73.65 (9.37) 254	131.42 (21.17) 223 74.48 (10.77) 223
LDL (mmol/L)	2.24 (0.83) 231	2.19 (0.85) 209	2.38 (0.83) 212	2.39 (0.84) 217
Triglycerides (mmol/L)	1.73 (0.94) 232	1.73 (0.89) 212	1.70 (0.88) 215	1.73 (0.94) 222
HbA1c (%)	6.37 (1.22) 240	6.32 (1.34) 212	6.27 (1.07) 254	6.38 (1.54) 223
HbA1c category (*n*) Normal (<5.7%) Prediabetes (5.7% to 6.4%) Diabetes (>6.4%)	65 (27.1%) 106 (44.2%) 69 (28.8%)	72 (34.0%) 80 (37.7%) 60 (28.3%)	62 (24.4%) 135 (53.1%) 57 (22.4%)	64 (28.7%) 106 (47.5%) 53 (23.8%)
BMI	33.82 (6.24) 240	33.31 (5.88) 215	35.22 (7.14) 254	35.09 (7.18) 223
Diabetes medication (*n*) ^††^	52 (21.8%)	36 (16.7%)	55 (22.1%)	41 (18.4%)

Data are mean (SD) and *n*, except for diabetes category and medications, which are *n* (%).

^†^Some follow-up biometric data are missing due to occasional difficulties taking accurate measurements (device failures caused by ambient temperature or other unknown causes, injury preventing proper application of blood pressure cuff, etc.).

^††^Diabetes medication use is based on reporting taking any one or a combination of metformin, insulin, or glimepiride. Baseline percentages reflect 7 participants (2 Lifestyle Africa and 5 usual care) not included in denominator due to missing data.

BMI, body mass index; HbA1c, Hemoglobin A1c; LDL, low-density lipoprotein.

The modal number of sessions held by CHWs across clubs was 17 (range of 10 to 17). Mean attendance across all sessions varied by cluster, ranging from 44% to 83%, with a mean of 61%. Across all clusters, 42% of participants attended at least 75% of the sessions held. Fidelity ratings based on observation of 74% of sessions indicated that mean completion rates of session facilitation elements (i.e., fully completed versus partially or not completed) for each club ranged from 62% to 95% (mean = 85.4%). Mean ratings on a 7-point Likert scale (1 = poor/never and 7 = excellent/always) of group facilitation skills, MI skills, and overall performance were 5.5 (range = 4.9 to 6.3), 5.4 (range = 4.7 to 6.2), and 5.5 (range = 4.7 to 6.3), respectively. There were no unanticipated problems and serious adverse events were rare, unrelated to the intervention, and similar across groups (11 in Lifestyle Africa versus 13 in usual care).

Empty models for the intent-to-treat analyses revealed club-level ICCs that ranged from 0.01 to 0.05 and repeated measures ICCs within person that ranged from 0.39 for systolic blood pressure to 0.98 for weight. The intent-to-treat analyses revealed the primary outcome of weight did not differentially change over time across conditions [time by condition interaction effect of *F*(1, 897) = 0.64, *p* = 0.42]. However, HbA1c did show differential change over time across conditions [time by condition interaction of *F*(1, 899) = 8.59, *p* = 0.004] with a Cohen’s *d* effect size of 0.21. None of the other secondary outcomes showed differential change over time: systolic blood pressure, *F*(1, 896) = 0.82, *p* = 0.37; diastolic blood pressure, *F*(1, 896) = 0.42, *p* = 0.52.; triglycerides, *F*(1, 846) = 0.17, *p* = 0.68; or LDL, *F*(1, 839) = 0.28, *p* = 0.60.

For the analysis with outcomes at the end of year 1 as the dependent variable, club level ICCs were 0.05 or less, although they were slightly higher at 0.07 and 0.09 for systolic and diastolic blood pressure, respectively. Table 3 provides the parameter estimates from the models that included baseline values for outcomes (except for percent weight change) and study arm. Means (adjusted for the baseline value of the variable and clustering) for each treatment arm, the estimate of the mean difference between groups, and the 95% confidence interval (CI) for each are presented. Mean weight change was −0.61% (95% CI = −1.22, −0.01) in Lifestyle Africa and −0.44% (95% CI = −1.06, 0.18) in usual care, with the between-group model–adjusted difference not statistically significant (= −0.17%; 95% CI −1.04, 0.71; *p* = 0.71). Absolute mean weight at follow-up was 84.13 kg (95% CI 83.61, 84.65) in Lifestyle Africa and 84.40 kg (95% CI 83.88, 84.91) in usual care, with the between-group model–adjusted difference not statistically significant (= −0.27; 95% CI −1.00, 0.47). There were also no significant differences between treatment arms in blood pressure, LDL cholesterol, or triglycerides. However, there was a significant difference in HbA1c at follow-up between arms (mean difference = −0.24%, Hedges’ *g* = 0.23, *p* = 0.001), with Lifestyle Africa participants averaging 6.23% (95% CI = 6.12, 6.34) and usual care averaging 6.47% (95% CI = 6.37, 6.58). Adding baseline BMI as a covariate to the models did not change the significance of the effect of treatment arm for any of the outcomes. We also considered whether HbA1c improvements might have been due to increases in medication use. However, results were essentially unchanged after removing from the analysis the 48 participants who had a reduction in HbA1c and were on diabetes medication (see Table 3). A tipping point analyses was used to examine the impact of missing data at year 1 follow-up on HbA1c effects. Results indicated that the effect of treatment on HbA1c would still be significant (*p* = 0.04) if Lifestyle Africa participants with missing data at the year one follow-up had an increase in HbA1c that was 6 times that of the mean increase in the control group (i.e., an increase of 0.78%) with a resulting usual care mean of 6.45% and a Lifestyle Africa mean of 6.31%.

**Table 3:** ANCOVA model summaries for each outcome.

	ICC	Lifestyle Africa	Usual care	Group difference	SE	*F* Test	*p*
**Basic models—No added covariates**							
% Weight change	0.026	−0.61 (−1.22, 0.01)	−0.44 (−1.06, 0.18)	−0.17 (−1.04, 0.71)	0.44	*F*(1,410) = 0.14	0.71
Weight	0.014	84.13 (83.61, 84.65)	84.40 (83.88, 84.91)	−0.27 (−1.00, 0.47)	0.37	*F*(1,409) = 0.51	0.48
Systolic BP (mm Hg)	0.097	129.56 (125.68, 133.45)	130.92 (126.94, 134.91)	−1.36 (−6.92, 4.21)	2.83	*F*(1,408) = 0.23	0.63
Diastolic BP (mm Hg)	0.062	74.23 (72.31, 76.16)	74.39 (72.42, 76.36)	−0.15 (−2.91, 2.60)	1.40	*F*(1,408) = 0.01	0.91
LDL (mmol/L)	0.022	2.27 (2.18, 2.35)	2.34 (2.25, 2.42)	−0.07 (−0.19, 0.05)	0.06	*F*(1,358) = 1.29	0.26
Triglycerides (mmol/L)	0.004	1.75 (1.63, 1.88)	1.78 (1.65, 1.90)	−0.02 (−0.20, 0.16)	0.09	*F*(1,369) = 0.07	0.80
HbA1c (%)	0.044	6.23 (6.12, 6.34)	6.47 (6.37, 6.58)	−0.24 (−0.39, −0.09)	0.08	*F*(1,406) = 10.32	0.001
**BMI as added covariate**							
% Weight change	-	−0.65 (−1.27, −0.04)	−0.40 (−1.01, 0.21)	−0.26 (−1.13, 0.62)	0.44	*F*(1,409) = 0.33	0.57
Weight	-	84.13 (83.61, 84.64)	84.40 (83.88, 84.92)	−0.27 (−1.01, 0.46)	0.37	*F*(1,408) = 0.54	0.46
Systolic BP (mm Hg)	-	129.49 (125.58, 133.39)	130.99 (126.98, 134.99)	−1.50 (−7.10, 4.10)	2.85	*F*(1,407) = 0.28	0.60
Diastolic BP (mm Hg)	-	74.10 (72.15, 76.06)	74.50 (72.50, 76.50)	−0.39 (−3.20, 2.41)	1.43	*F*(1,407) = 0.08	0.78
LDL (mmol/L)	-	2.27 (2.19, 2.35)	2.33 (2.24, 2.42)	−0.06 (−0.18, 0.06)	0.06	*F*(1,357) = 1.01	0.32
Triglycerides (mmol/L)	-	1.75 (1.62, 1.87)	1.78 (1.65, 1.91)	−0.03 (−0.21, 0.15)	0.09	*F*(1,368) = 0.31	0.72
HbA1c (%)	-	6.23 (6.12, 6.33)	6.47 (6.37, 6.58)	−0.25 (−0.40, −0.10)	0.08	*F*(1,405) = 10.52	0.001
**Excluding participants on diabetes medication with reductions in HbA1c (*n* = 48)**							
HbA1c (%)	-	6.20 (6.11, 6.30)	6.37 (6.28, 6.45)	-0.16 (-0.29, -0.03)	0.07	*F*(1,358) = 5.86	0.02

Means and (95% CIs). Means are adjusted means controlling for baseline.

BMI, body mass index; BP, blood pressure; CI, confidence interval; HbA1c, hemoglobin A1c; ICC, intraclass correlation coefficient; LDL, low-density lipoprotein; SE, standard error of the difference.

Analyses of effects within sex revealed no significant interaction effects for any of the outcomes (see Table 4). Treatment effects within sex indicated a slightly lower effect of treatment on HbA1c for men [group difference = 0.14%, 95% CI -0.33, 0.62; *t*(404) = 0.60, Hedges’ *g* = 0.11, *p* = 0.55] than for women [group difference = 0.25%, 95% CI 0.10, 0.41; *t*(404) = 3.33, Hedges’ *g* = 0.25, *p* = 0.002]. Analysis of effects on weight change within diabetes categories revealed no significant interaction effect [*F*(2, 405) = 0.49, *p* = 0.62].

**Table 4:** ANCOVA models separated by sex.

	Male*				Female					
	Group difference	SE	*t* Test	*p*	Group difference	SE	*t* Test	*p*	Interaction test	*p*
% Weight loss	0.11 (−2.21, 2.44)	1.18	*t*(408) = 0.10	0.92	0.17 (−0.73, 1.08)	0.46	*t*(408) = 0.38	0.71	*F*(1, 408) = 0.01	0.96
Systolic BP (mm Hg)	4.26 (−7.15, 15.64)	5.80	*t*(406) = 0.73	0.46	1.04 (−4.61, 6.70)	2.88	*t*(406) = 0.36	0.72	*F*(1, 406) = 0.32	0.57
Diastolic BP (mm Hg)	1.53 (−4.37, 7.42)	3.00	*t*(406) = 0.51	0.61	0.004 (−2.81, 2.82)	1.43	*t*(406) = 0.01	0.99	*F*(1, 406) = 0.27	0.61
LDL (mmol/L)	−0.07 (−0.46, 0.31)	0.20	*t*(356) = 0.36	0.72	0.09 (−0.04, 0.21)	0.07	*t*(356) = 1.29	0.20	*F*(1, 356) = 0.58	0.45
Triglycerides (mmol/L)	0.21 (−0.28, 0.69)	0.25	*t*(367) = 0.84	0.40	0.003 (−0.18, 0.19)	0.09	*t*(367) = 0.03	0.97	*F*(1, 367) = 0.64	0.43
HbA1c (%)	0.14 (−0.33, 0.62)	0.24	*t*(404) = 0.60	0.55	0.25 (0.10, 0.41)	0.08	*t*(404) = 3.18	0.002	*F*(1, 404) = 0.19	0.67

Means and (95% CIs). Parameter estimates are from a single model for each outcome where the F test is for the sex by treatment group interaction and the *t* test is for the differences between arms within each sex.

*Subgroup *n*’s for % weight loss are 22 (male control), 22 (male treatment), 201 (female control), and 193 (female treatment). Subgroup *n*’s for Systolic and Diastolic BP are 22 (male control), 21 (male treatment), 201 (female control), and 191 (female treatment). Subgroup *n*’s for LDL are 18 (male control), 20 (male treatment), 166 (female control), and 183 (female treatment). Subgroup *n*’s for triglycerides are 19 (male control), 21 (male treatment), 173 (female control), and 185 (female treatment). Subgroup *n*’s for HbA1c are 22 (male control), 21 (male treatment), 201 (female control), and 191 (female treatment).

BP, blood pressure; CI, confidence interval; HbA1c, hemoglobin A1c; LDL, low-density lipoprotein; SE, standard error of the difference.

To analyze the clinical meaningfulness of the HbA1c effect, we categorized HbA1c values into normal (<5.7%), prediabetic (5.7% to 6.4%), and diabetic (>6.4%) categories and determined if improvements or deteriorations in diabetes category differed by group. In the control arm, 8% (4/49) of participants improved from the diabetes category and 13% (16/119) improved from the prediabetes category. In the Lifestyle Africa arm, 14% (9/65) of participants improved in the diabetes category, and 26% (24/93) improved in the prediabetes category. In the control arm, 13% (7/55) of participants deteriorated from the normal category, and 6% (7/119) deteriorated from the prediabetes category, whereas in the Lifestyle Africa arm 11% (6 of 54) deteriorated from the normal category, and 4% (4/93) deteriorated from the prediabetes category. Participants in the Lifestyle Africa arm had a higher probability of lowering (improving) their HbA1c by at least 1 category and a lower probability of raising their diabetes category (odds ratio [OR] = 1.52, 95% CI 1.04, 2.22, *p* = 0.03).

## Discussion

The Lifestyle Africa intervention was generally successfully delivered, and more than 40% of participants attended 75% or more of the sessions. While the intervention did not have a significant effect on the primary outcome of weight loss or the secondary indicators of blood pressure, LDL cholesterol, and triglycerides, the intervention had a significant positive effect on HbA1c. There was a mean decline in the intervention group and a mean increase in the control group with a mean HbA1c difference of 0.24%. This is approximately a quarter of the effect observed for metformin in randomized trials [38]. The effect was present for men and women, although the small number of men in the study hinders firm conclusions for men. Analysis using HbA1c-defined clinical diabetes categories indicated that the intervention group had nearly twice the odds of improving their diabetes category (i.e., moving from diabetes to prediabetes or prediabetes to normal).

Results are fairly consistent with the 2 prior studies conducted in LMIC countries that used a peer support model of treatment delivery and found some positive intervention effects [16,17]. The Kerala DPP had outcomes that consistently favored the intervention arm and the strongest effects on systolic blood pressure, triglycerides, and HbA1c, although none of these effects were statistically significant [16]. The study in Grenada [17] reported somewhat positive effects based on a nonlaboratory-based cardiovascular health index that included blood pressure and weight. In the present study, outcomes also consistently favored Lifestyle Africa, but only HbA1c effects were statistically significant. To our knowledge, our study is the first of its kind to show a significant effect on a biologic outcome, i.e., HbA1c.

Considering the positive effect of Lifestyle Africa on HbA1c, the lack of an effect on the primary outcome of weight loss was unexpected as blood glucose changes tend to be associated with weight loss [39–42]. However, our result is similar to the Kerala DPP study, which also found that the effect on HbA1c was stronger than on weight [16]. One explanation is that HbA1c changes may be related to dietary changes or changes in physical activity independent of weight loss [43,44].

With respect to intervention feasibility, results indicated that CHWs were able to deliver the sessions and that participants attended at acceptable levels. In almost all clubs, 16 or 17 sessions were held. Mean attendance was 61% across all sessions and all clubs, and 42% of participants attended at least 75% of the sessions. This is very consistent with delivery of 96% of planned sessions and median attendance of 60% observed in the peer-led Kerala DPP study [16]. These levels of attendance in resource-constrained environments compare well with attendance levels observed for the DPP in the US where less than 50% of participants attend all 16 core sessions [45]. Taken together with the quite high ratings of fidelity to the intervention, our findings support the feasibility of the Lifestyle Africa approach.

Although our results are similar to the prior studies utilizing nonprofessional interventionists in middle-income countries, the present study differs in some important ways. First, participants were mostly female, older rather than middle-aged, and mostly unemployed or receiving a pension rather than working. Following and engaging in a novel program and making changes in long-established behavior patterns may be more difficult for older participants who are experiencing both physical and cognitive decline. In addition, weight loss might have been hampered by a cultural norm of obesity among women in this population [46]. Second, the program was delivered entirely by lower resource CHWs, whereas the Kerala DPP utilized research program staff [16], a local resource provider, and expert panel members comprised of specialist advisors on diabetes, nutrition, and physical activity who provided education during some of the sessions. Use of video as a tool to provide all expert content and guide session flow in Lifestyle Africa may be particularly valuable in settings where use of these additional high resource personnel is not feasible. Video-based programs can also be relatively easily adapted to new languages, cultures, and contexts.

Strengths of the study include a rigorous cluster randomized design, a good level of participant attendance at sessions, and high levels of participant follow-up. These strengths are particularly notable in light of the daily challenges CHWs and participants face in this low-resource setting. Limitations of the study include the lack of a rigorous dietary intake measure, such as a 24-hour food recall, which could have identified any changes in dietary patterns that may have been related to HbA1c changes. Similarly, rigorous measures of physical activity and diabetes medication adherence would have been desirable. In addition, field-based measurements may include more error than clinical- and laboratory-based measures. Lipids were also assessed without fasting as this was judged to be too burdensome for participants who might have been experiencing food insecurity. Mean follow-up times were 7.5 to 14 days earlier for control participants than for Lifestyle Africa participants. This difference is unlikely to have had an impact on the observed HbA1c difference between groups as HbA1c reflects mean blood sugar levels over the prior 3 months. Although loss to follow-up was low overall, there was some indication that male sex, higher weight, and lower HbA1c were associated with a greater likelihood of dropping out of the intervention. Findings based on our sample of predominantly older, retired females of lower socioeconomic status should be generalized cautiously to other demographic groups and settings.

The results of this study, taken together with other early trials in LMICs are mixed, and thus how we proceed moving forward with these types of interventions are not clear-cut and may depend on what lens is used. On the one hand, both the current study as well as the Kerala study in India [16] did not achieve clinically significant weight loss, and the impact on HbA1c in our study was clinically modest, with no other significant outcomes in these 2 studies. These modest outcomes raise questions about the utility of continuing these lifestyle intervention adaptions in LMICs given that they can be costly and time consuming.

On the other hand, this study, the Kerala study [16], and the Grenada study [17] were nonetheless shown to be feasible with regard to use of local lay interventionists, recruiting and retaining study participants, and intervention engagement. While intervention development costs are significant, these instances incorporated several cost efficiencies such as use of lay interventions and group delivery of the intervention. Rather than jettisoning this approach entirely, perhaps improvement in intervention content and strategy, enhancing the dose of treatment, and other intervention delivery factors could lead to significant impact using this overall paradigm. For example, increasing “hands-on” activities such as physical activity and food preparation to allow practice of behavioral skills in session and thereby reduce complete reliance on self-regulation outside of sessions may be helpful. With regard to enhancing dose, longer interventions that provide ongoing support for necessary lifestyle changes (e.g., access and exposure to fresh fruits and vegetables) in settings where environmental barriers (e.g., food insecurity) are great may be worth testing.

In conclusion, results from this study indicate that the Lifestyle Africa intervention is feasible and had a clinically modest impact on HbA1c. The video-based design that avoids the need for expert involvement is highly scalable and could also be delivered via smart phone or an online platform. Before the intervention can be recommended for widespread scale-up, further studies are needed to confirm the feasibility of the intervention and generalizability of the findings to other countries and settings. In addition, cost-effectiveness analyses are important to show the feasibility and value of scale-up in LMICs.

## Supporting information

S1 Checklist CONSORT Checklist. CONSORT, Consolidated Standards of Reporting Trials.
(DOCX)

## Abbreviations

BMI: body mass index
CHW: community health worker
CI: confidence interval
CONSORT: Consolidated Standards of Reporting Trials
CVD: cardiovascular disease
DPP: Diabetes Prevention Program
HbA1c: hemoglobin A1c
HDL: high-density lipoprotein
ICC: intracluster correlation coefficient
LDL: low-density lipoprotein
LMIC: low- and middle-income country
MI: motivational interviewing
NGO: nongovernmental organization
OR: odds ratio
T2DM: type 2 diabetes mellitus
